# The lignin deposition responses of different poplar root systems to salt stress

**DOI:** 10.3389/fpls.2026.1888861

**Published:** 2026-09-22

**Authors:** Jia-Hui Meng, Wen-Teng Zuo, Lei Rao, Kang-Ping Liao, Hong-Chao Liu, Liu-Qiang Wang, Ting-Ting Sun

**Affiliations:** 1 College of Horticulture, China Agricultural University, Beijing, China; 2 State Key Laboratory of Tree Genetics and Breeding, Key Laboratory of Tree Breeding and Cultivation of the State Forestry Administration, Research Institute of Forestry, Chinese Academy of Forestry, Beijing, China

**Keywords:** casparian strip, lignin, poplar, root, salt stress

## Abstract

Salt stress significantly constrains plant growth and development, with the root system serving as the primary organ for sensing and responding to salinity. Poplar (*Populus* spp.), a widely cultivated tree species, plays a pivotal role in urban greening, windbreak and sand fixation, and various economic sectors. Six poplars as experimental materials to analyze lignin deposition in the root system and variations in the casparian strip under salt stress. Lignin enrichment and changes in the casparian strip are as follows: *P. alba × P. glandulosa* ‘84K’ showed the most pronounced changes, followed by *P. cathayana* and *P. tremula × P. alba*, then *P*. *deltoids* × *P*. *euramericana* ‘Nanlin895’, *Populus* × *euramaricana* ‘Bofeng 3 hao’ and *P. simonii.* Transcriptome sequencing revealed the molecular mechanisms in the 84K poplar and *P. simonii* with the greatest and smallest lignin increase, respectively. The results reveal that 84K poplar and *P. simonii* mainly regulate the accumulation of lignin through phenylalanine metabolism, starch and sucrose metabolism, as well as the MAPK signaling pathway under salt stress. This study provides critical theoretical support for elucidating the molecular response mechanisms of root lignin and the casparian strip under salt stress.

## 1 Introduction

Soil salinity is a primary abiotic stress limiting agricultural and silvicultural productivity, with salinization presenting an escalating challenge on a global scale. Salt stress exerts a detrimental influence on plant growth and development, especially regarding root development. Roots are the first line of defense against soil salinity, by perceiving and reacting to salt levels, they play a fundamental role in how plants survive high-salinity environments (van Zelm et al., 2023; Tang et al., 2024).

Lignin acts as a complex, high-molecular-weight phenolic polymer that is both hydrophobic and structurally heterogeneous. By promoting the thickening of secondary cell walls, it provides essential mechanical stability and impermeability, allowing plants to maintain rigidity and defend against various abiotic stresses (Liu et al., 2018). Lignin metabolism also plays an important role in plant growth and stress tolerance (Cesarino, 2019). Many plants accumulate lignin under salt stress, such as corn increases its lignification level to resist salt stress (Oliveira et al., 2020). Increased lignin accumulation under salt stress has been documented in various species, including *Populus canescens* and *Populus euphratica* (Janz et al., 2012), a similar induction of lignification has also been identified in tomato (Chattha et al., 2024). Lignin is synthesized by plant cells’ phenylalanine/tyrosine metabolic pathway, plants synthesize lignin through the polymerization of various enzymes, such as through the action of several enzymes notably, phenylalanine ammonia-lyase (PAL), cinnamate 4-hydroxylase (C4H), *p*-coumarate 3-hydroxylase (C3H), *p*-hydroxycinnamoyl CoA: shikimate *p*-hydroxycinnamoyl transferase (HCT), cinnamate 4-hydroxylase (4CL), caffeoyl-CoA *O*-methyltransferase (CCoAOMT), cinnamoyl-CoA reductase (CCR), caffeic acid *O*-methyltransferase (COMT), and cinnamyl alcohol dehydrogenase (CAD) (Ge et al., 2023; Swetha et al., 2024).

The casparian strip serves as a specialized hydrophobic boundary within the plant root. By utilizing a lignin-based polymer, it obstructs the apoplastic pathway to ensure the precise sorting of ions and nutrients entering the stele (Wang et al., 2019). The casparian strip is a specialized cell wall of the inner cortical cells of plant roots, which is mainly involved in water and mineral transport between the cortex and the vascular column (Kalmbach et al., 2017), and is also capable of controlling the selective uptake of ions by the cells, as well as having a sequestering effect on water and minerals in the vascular system (Gao et al., 2023). Maize shows the development of casparian strip under high saline conditions (Wang et al., 2022). In unfavorable environments, plants form casparian strips earlier to resist stress (Chen et al., 2011), and salt-tolerant plants have an earlier period of casparian strip formation and deeper corking (Chen et al., 2024). Under salt stress, casparian strip form earlier in the rice root system, and the location of the casparian strip is closer to the roots, and the width of the casparian strip increases (Krishnamurthy et al., 2009).

Evolutionary adaptations to salt stress in plant roots involve multiple pathways; notably, phenylalanine metabolism, starch and sucrose metabolism, and MAPK signaling collectively contribute to enhanced salt tolerance. The grapevine roots undergo enrichment in lignin biosynthesis and sugar metabolism under salt stress (Chen et al., 2025). The phenylalanine metabolism and starch and sucrose metabolism were active for energy homeostasis to cope with salt stress in cotton roots (Chen et al., 2016). An upregulated expression of sugar reducing and starch synthesis and degradation genes was also observed in the roots of *T. chinensis* (Li et al., 2023). Starch and sucrose metabolism and galactose metabolism were the key metabolic pathways in the salt tolerance in water spinach (Li et al., 2025). The MAPK cascade represents a highly conserved signaling mechanism across eukaryotic species, serving as a pivotal regulator of plant growth, maturation, and responses to diverse environmental pressures. Transcriptomic analysis by Li et al. (2024) underscored this, identifying the MAPK pathway as the most significantly enriched signaling route in salt-stressed foxtail millet. Similarly, research on tomatoes has highlighted that differentially expressed genes (DEGs) are predominantly associated with amino acid biosynthesis, carbon metabolism, and notably, the MAPK signaling framework (Wei et al., 2022).

Poplar is an important tree species for greening planting in the northern of China. Researching salt tolerance of poplar and its mechanism is of great significance for the improvement of plant salt tolerance and the restoration of saline-alkali land. In this study, six kinds of poplar root systems were used as test materials, and the differences in the response of root systems to lignin deposition under salt stress were compared by staining with resorcinol, determining the lignin content, observing the changes in the position of casparian strips, and observing the lignin deposition in the root systems after salt stress by using stimulated raman scattering microscopy (SRS) technology. Afterward, the lignin metabolism mechanism of the root system of different kinds of poplar under salt stress was further analyzed by transcriptome sequencing analysis, and the genes related to lignin deposition were excavated, which provided critical theoretical support for elucidating the molecular response mechanisms of root lignin and the casparian strip under salt stress.

## 2 Materials and methods

### 2.1 Plant materials and growth conditions

The study materials consisted of six poplar species, including *Populus alba* × *Populus glandulosa*, *Populus tremula × Populus alba*, *Populus cathayana*, *Populus simonii*, *Populus* × *euramaricana* ‘Bofeng 3 hao’ and *Populusdeltoids* × *Populuseuramericana* ‘Nanlin895’. The above material designations are 84K, 717, QY, XYY, BF3, and NL895, respectively.

The poplars were grown on a solid medium in a phytotron (25°C, 16/8 h light/dark, and 50 μM/m²/s light intensity). Subsequently, the seedlings were transplanted into hydroponic conditions, where the temperature of the growing room was maintained at 22°C and the relative humidity ranged from 70% to 75%. The seedlings grew for 60 days, and the nutrient solution (Coolaber, NS10115, Beijing, China) was replaced three times a week while the pH of the growth conditions was kept between 5.8 and 6.2. Each experiment was conducted with three independent replications.

### 2.2 Stress treatments

To evaluate the salt stress tolerance of different kinds of poplar, poplars grown for 60 days in hydroponic conditions were treated with 0 (control, CK) and 75 mM NaCl (salt stress, ST) solution for 3 days (Song et al., 2024), and plants were harvested and sampled. The root samples were instantly frozen in liquid nitrogen at -80 °C for biochemical analysis. Different growth parameters, as well as ionic and physiological indices, were recorded accordingly.

### 2.3 Histochemical staining and root anatomy

After salt stress treatment, 0–1 cm poplar root apices were sectioned using a vibrating blade microtome (VT1000s; Leica, Wetzlar, Germany). Poplar root tips from 0 to 1 cm were sectioned at a thickness of 50 μm per slice, each group consists of 20 pieces, which is 1 mm. The root system can be divided into 10 groups with a gradient of 1 mm. The root apices were stained with Berberine Sulfate (0.2%) for 1 h under dark conditions, then rinsed three times with deionized water, and then randomly selected sections were stained with Toluidine Blue O (0.1%), and the positions of casparian strip were observed with a fluorescence microscope (OlympusBx51, Japan).

In Phloroglucinol HCl staining, using the above method to cut off 5 cm of the root apices, the cross-section was treated with 37% HCl for 30 s and then stained with 1.0% (w/v) phloroglucinol for 30 s.

### 2.4 Determination of lignin content

Lignin analyses were performed using a lignin content detection Kit (Boxbio, Beijing, China) according to the manufacturer’s instructions. The solution was measured at 280 nm using SpectraMax^®^ Paradigm, and the lignin content was calculated according to the absorbance (Wang et al., 2025).

### 2.5 SRS microscopy

Transverse sections of 30 μm were cut freehand at a distance of 10 cm from the roots using a razor blade, and transverse sections at the 10 cm position of the root system of different poplar species were analyzed by SRS, and lignin autofluorescence imaging was observed on root transverse sections of the control and salt-stress-treated groups (Man et al., 2018).

### 2.6 Transcriptome sequencing

The roots of 0.0-0.5 cm in different kinds of poplar, which are control and salt-treated, were selected, and total RNA was extracted from the tissues by the trizol method. The concentration and purity of the total RNA extracted were examined using nanodrop, the integrity of RNA was detected by agarose gel electrophoresis, and the RIN value was determined by Agilent Technologies 2100 bioanalyzer; mRNA was enriched with magnetic beads with Oligo (dt) and used to analyze transcriptome information. mRNA molecules were purified and then fragmented into small pieces for cDNA synthesis. The synthesized cDNA was subjected to end-repair and then was 3’ adenylated. Adapters were ligated to the ends of these 3’ adenylated cDNA fragments. After the PCR reaction, PCR products were purified and dissolved in EB solution. The library was validated on an Agilent Technologies 2100 bioanalyzer, followed by sequencing using the Illumina sequencing platform. Transcriptome analysis was performed by Biomarker Technologies (Beijing, China). After rapid filtering (version 0.18.0), the HISAT2.2.4 and Bowtie2 tools were used to compare clean reads with the poplar genomes (https://phytozome-next.jgi.doe.gov/info/ptrichocarpa_v4_1) and (https://doi.org/10.6084/m9.figshare.12369209). RESM software was used to calculate the values of fragment per kilobase of transcript per million mapped reads (FPKM). DEGs were determined according to cutoffs of log2(fold change)≥1 and p ≤ 0.05. Kyoto Encyclopedia of Genes and Genomes (KEGG) and Gene Ontology (GO) tools were used to analyze the DEGs. Quantitative real-time PCR (qRT-PCR) analysis was conducted according to Sun et al. (2021), and the primers used for qRT-PCR are listed in **Supplementary Table 1**.

### 2.7 Statistical analysis

The histogram data were normalized. Statistical analysis was performed using one-way analysis of variance (ANOVA). The significant differences between treatment means were determined using Duncan’s multiple range test (DMRT) at a significance level of *P* < 0.05.

## 3 Results

### 3.1 Response of lignin synthesis to salt stress

To quantitatively evaluate the response of lignin deposition to salt stress, we first measured the lignin content in the roots of six poplar clones before and after NaCl treatment. The relative lignin content increased in all clones under salt stress, but the magnitude of this increase varied significantly among genotypes. 84K exhibited the most substantial increase, with a relative lignin content of 1.78-fold compared to the control, indicating a highly responsive lignin biosynthesis pathway. In contrast, BF3 and XYY showed relatively weak responses, with increases of only 1.24-fold and 1.23-fold, respectively. The lignin contents of QY, 717, and NL895 increased by 1.62-, 1.47-, and 1.41-fold, respectively, indicating intermediate responses (**Figure 1A**).

**Figure 1:**
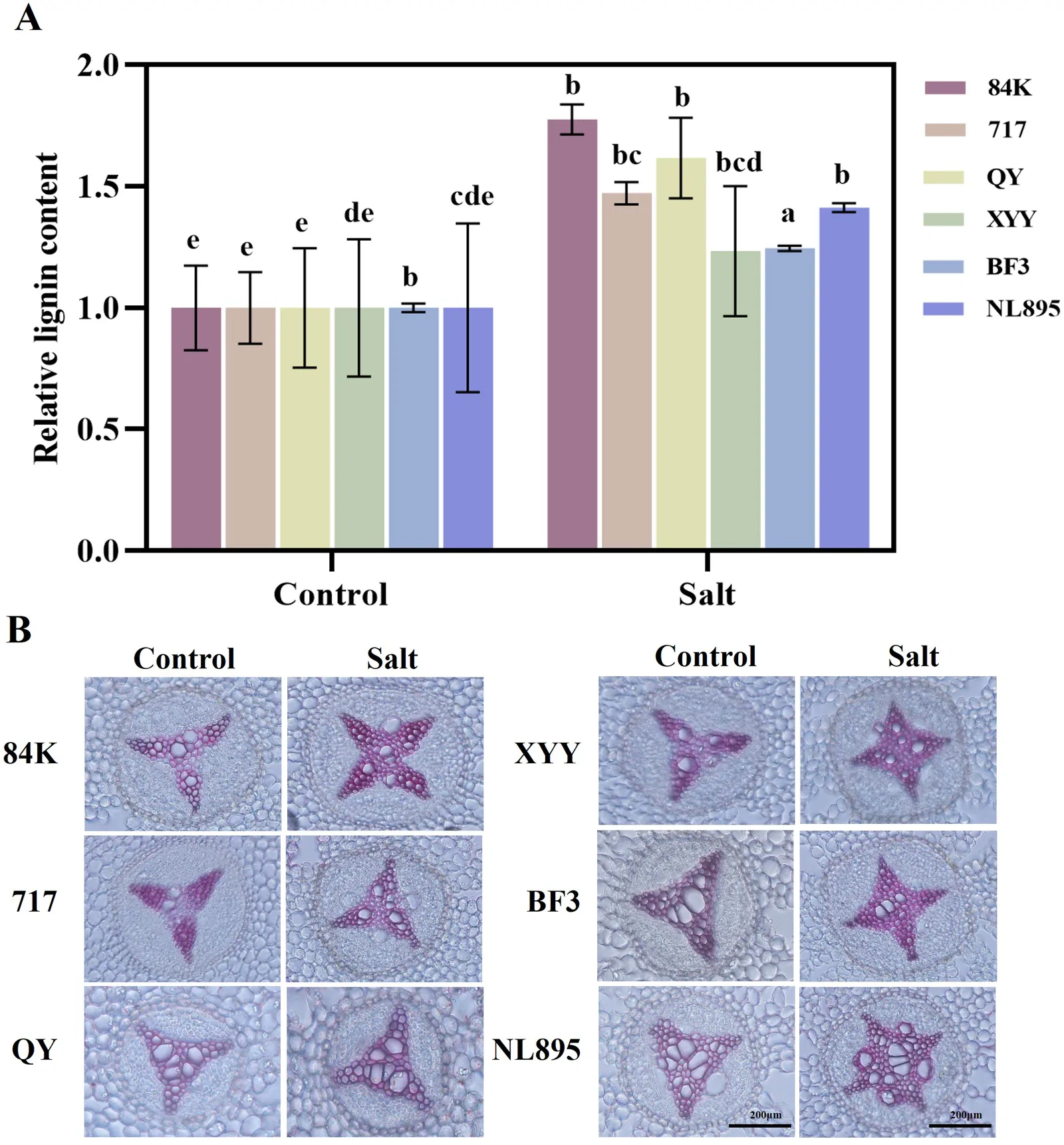
**(A)** Lignin content in root tips from 6 poplar species, with the data for the bar chart normalized. Different lowercase letters indicate significant differences in relative lignin content between the 6 poplar species before and after salt stress treatment (P < 0.05). Each staining was performed with 3 biological replicates, with no less than 15 root tips per replicate. **(B)** Phloroglucinol staining of root tips from 6 poplar species.

To visually examine the spatial distribution of this lignin accumulation was performed phloroglucinol-HCl staining on root cross-sections (**Figure 1B**). In agreement with the quantitative data, most clones displayed deeper red coloration after salt treatment, particularly in the vascular cylinder and xylem cells. Notably, the staining intensity varied among clones and did not strictly correlate with the quantitative fold-changes, reflecting the semi-quantitative nature of histological staining. Nevertheless, the images qualitatively support the conclusion that salt stress induces tissue-specific lignin deposition as an adaptive response to external stress, with the most pronounced visual difference observed in 84K and it also showed the highest quantitative increase.

### 3.2 Response of casparian strip formation to salt stress

Under salt stress, the casparian strip regulates the rational absorption of water and minerals, enabling cells to selectively take up ions. Consequently, under saline conditions, the casparian strip tends to develop earlier and forms closer to the root tip. In this study, the berberine-toluidine blue double staining method was employed to stain transverse sections of poplar root tips before and after salt treatment, allowing for the observation of changes in the position of casparian strip formation across six poplar clones.

**Figure 2A** shows the microscopic observation of a transverse section during the initial stages of casparian strip formation. The distances from the root tip to the point where the casparian strip first appears were statistically analyzed for the different poplar root systems, with the results presented in **Figure 2B**. Under control conditions, the casparian strip of BF3 was closest to the root tip, appearing within the 3.0–3.5 mm range, while that of 84K was the furthest, located approximately 6.0–6.5 mm from the tip.

**Figure 2:**
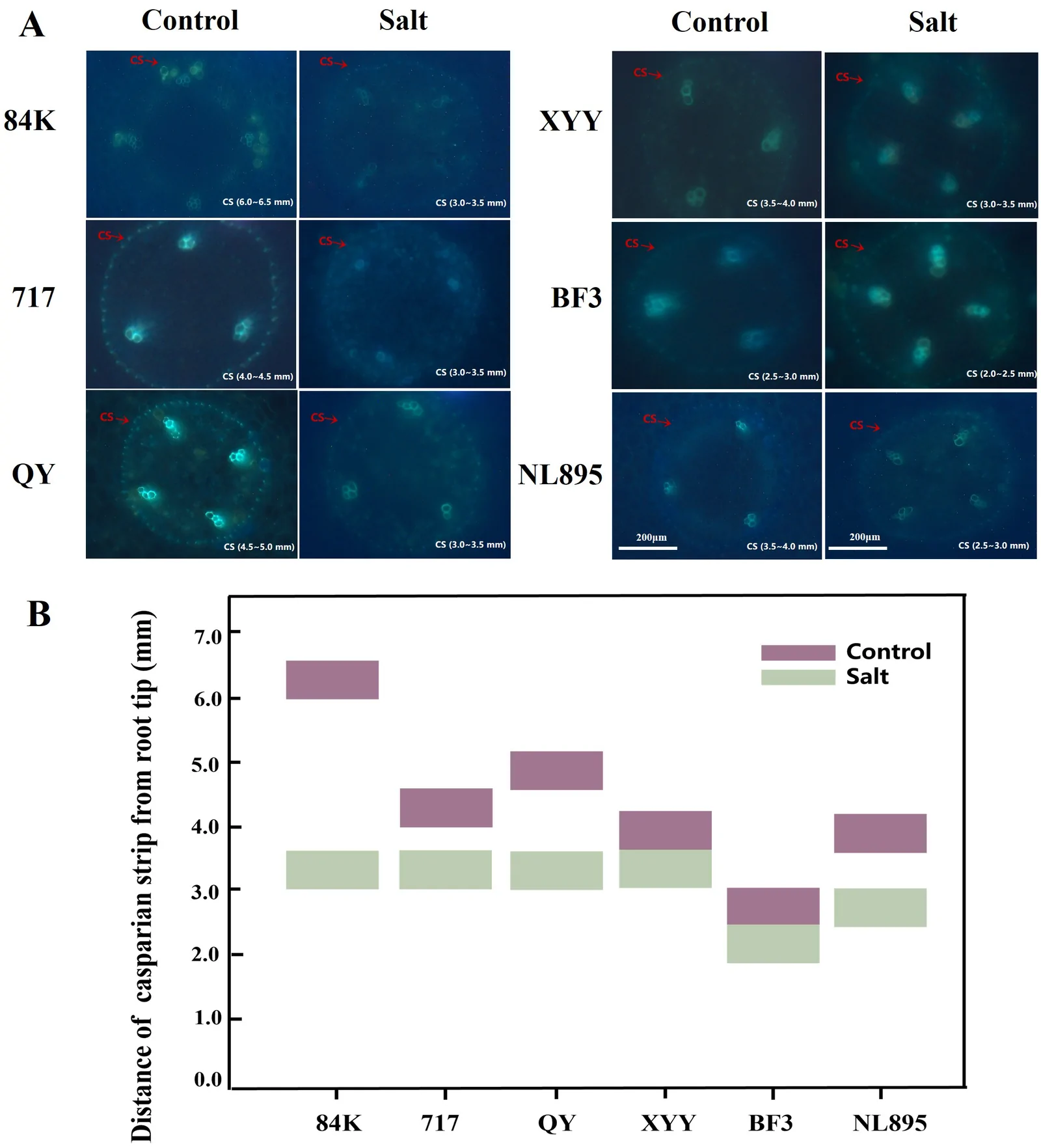
**(A)** Microscopic observation of casparian strips in root tips from 6 poplar species. **(B)** Position of casparian strips in root tips from 6 poplar species. Different lowercase letters indicate significant differences in distance to root tip between the 6 poplar species before and after salt stress treatment (P < 0.05). Each staining was performed with 3 biological replicates, with no less than 15 root tips per replicate.

Following salt stress treatment, the casparian strips in all six poplar varieties formed prematurely and closer to the root tip. For 84K, 717, QY and XXY, the casparian strips first appeared in the 3.0–3.5 mm range. Notably, 84K exhibited the most significant shift, with the casparian strip position moving 3.0 mm toward the root tip. The position in QY advanced by 1.5 mm, while 717 and NL895 both showed an advancement of 1.0 mm. In contrast, XYY and BF3 showed the least change, advancing by only 0.5 mm.

To evaluate the structural and biochemical responses to salinity, SRS imaging was employed to observe the root tip casparian strips (Xu et al., 2021). The SRS signal intensity of the casparian strip increased across different poplar varieties following salt treatment (**Figure 3A**). Analysis of the SRS spectra revealed a robust Raman signal at 1602 cm^-1^ for the aromatic deposition (including lignin and suberin) associated with casparian strip development of all poplar root tips (**Figure 3B**). Furthermore, BF3 exhibited higher basal aromatic deposition both before and after salt stress. Notably, 84K showed the most significant increase in aromatic deposition at the 10 cm mark following salt treatment, followed by QY and 717, the results are consistent with the lignin content.

**Figure 3:**
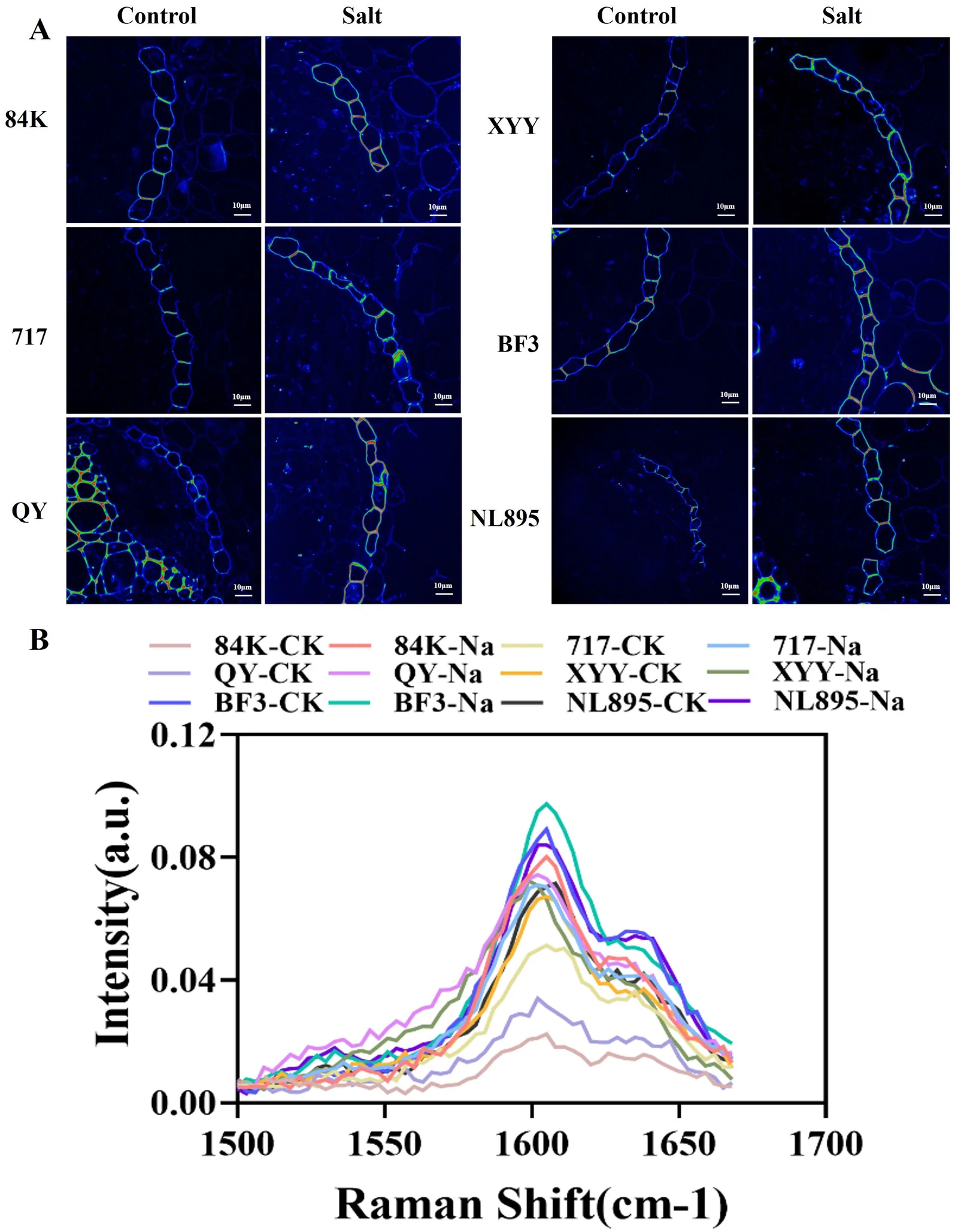
**(A)** SRS images of aromatic deposition (including lignin and suberin) associated with casparian strip development on root tips from 6 poplar species. Scale bar=10μm. **(B)** SRS spectra of aromatic deposition in root tips from 6 poplar species. Error bars represent ± SD. Each staining was performed with 3 biological replicates, with no less than 15 root tips per replicate.

### 3.3 Quality analysis of sequencing results from salt stress treatments in the root system of 84K and XYY

The average Q30 scores of RNA from 84K samples collected in the CK and salt stages and sequenced on the Illumina Hi-Seq platform after filtration were greater than 95.41%, and the XYY samples were greater than 94.45%. The GC content was approximately 44.28% and 44.10% in 84K and XYY. Data quality is reliable and can be used for subsequent analysis (**Supplementary Tables S2**, **S3**).

Transcriptome analysis of the root system of 84K under salt stress. Through transcriptome sequencing, the analysis of genes expression in the 84K control group and the salt-stressed root system revealed a total of 2015 significantly different genes. Among them, 1179 genes were up-regulated and 836 genes were down-regulated, accounting for 58.5% and 41.5% of the differentiating genes respectively (**Supplementary Figure 1**). Through the analysis of gene expression in the control and salt-stressed groups of XYY. A total of 2999 genes with significant differences were detected in Psi.CK-VS-Psi.Salt, of which 1659 genes were up-regulated and 1340 genes were down-regulated, accounting for 55.3% and 47.7% of the differentiated genes, respectively (**Supplementary Figure 2**). Those indicate that 84K and XYY mainly regulates genes expression through active stress response mechanisms to resist external salt stress and protect the plant from the damage caused by salt stress.

The ST and CK transcriptome data of 84K and XYY have been deposited into the NCBI database, with the bioproject accession number are PRJNA1494161 and PRJNA1494114. To confirm the findings of RNA-seq, we conducted expression analysis on 10 poplar genes through qRT-PCR in 84K and XYY poplars, respectively. The findings indicated no significant differences compared to the RNA-seq data, with both upregulated and downregulated genes exhibiting similar patterns (**Supplementary Figures S2**, **S3**). These results confirm the RNA-seq findings and effectively reflect the changes in the transcriptome noted in this study.

### 3.4 GO enrichment and KEGG functional of differential genes

To analyze the biological functions of DEGs, GO functional annotation of DEGs was carried out from three aspects: biological process, cellular component, and molecular function (**Figures 4A, C**). The GO annotations of DEGs were obtained. In biological processes, DEGs are mainly enriched in metabolic process, cellular process, biological regulation, and response to stimulus; most DEGs in cellular component are concentrated in the cellular anatomical entity, and intracellular. The molecular function annotated DEGs are mainly focused on binding and catalytic activity.

**Figure 4:**
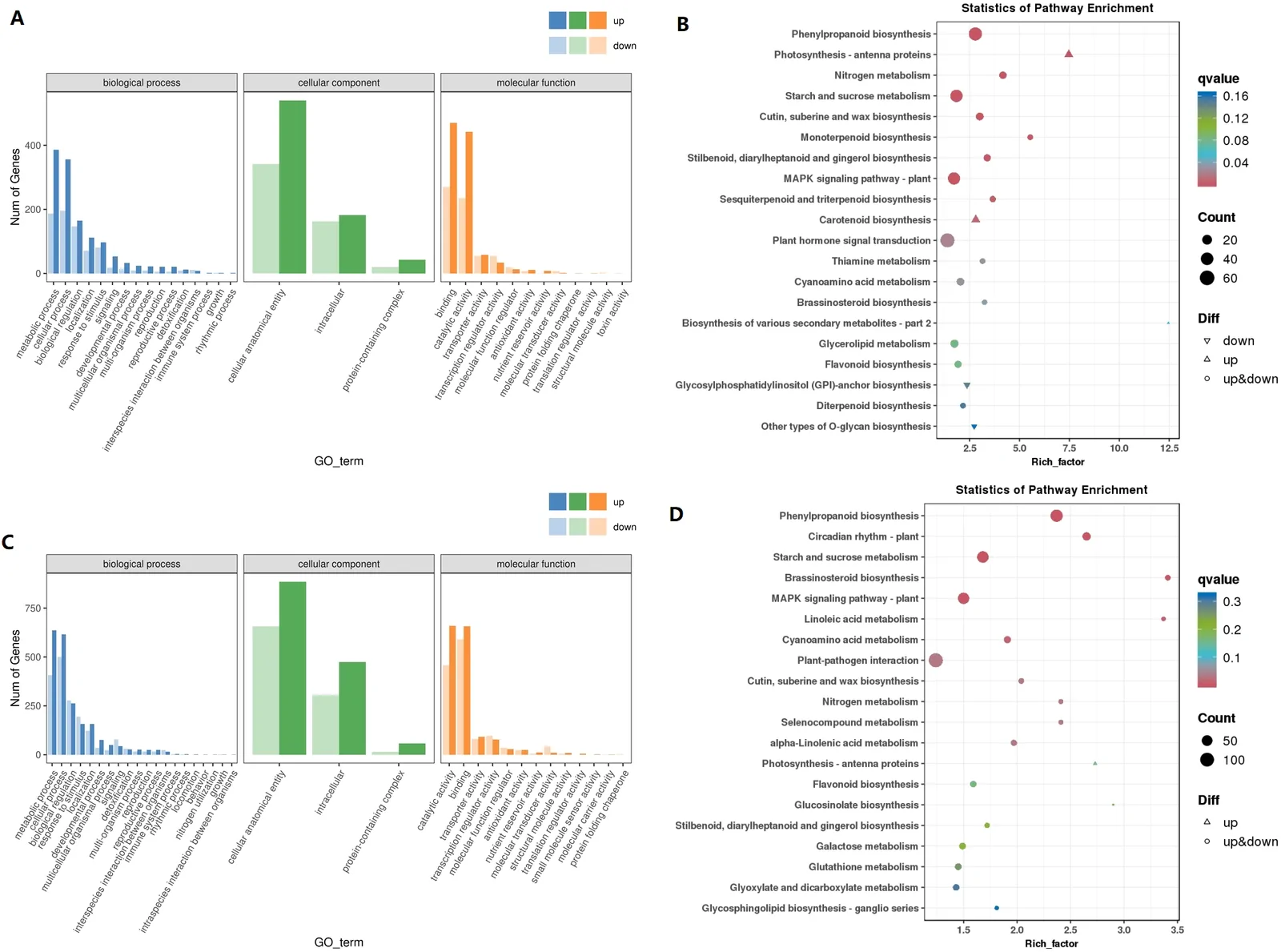
**(A, C)** GO functional annotation and **(B, D)** KEGG enrichment analysis of differential genes in 84K and XYY roots under salt stress.

After KEGG analysis of the differential genes of 84K and XYY under salt stress, the top 20 pathways with the highest degree of enrichment were selected and the enrichment bubble figure was drawn (**Figures 4B, D**).

### 3.5 The phenylpropanoid biosynthesis pathway in Populus roots under salt conditions

Under salt stress, the phenylpropanoid biosynthesis pathway is also the main enriched pathway in 84K and XYY. The expression of *COMT* (Pag.A17G000512 and Potri.012G006400) was increased in both 84K and XYY. The expression levels of *CCoAOMT* (Pag.A14G001068 and Pag.B14G001013) and peroxidase (*POD*, Pag.B01G003911) genes have all increased in 84K. The expression of *HCT* (Potri.004G017600, Potri.010G0053800, Potri.007G003800, Potri.0118G104700, Potri.010G0054002) gene increased in XYY. Most of *HCT* (Pag.A10G000724, Pag.A10G000746, Pag.B05G000312, Pag.B06G002549, Pag.B10G000454, Pag.B10G000457 and Pag.B17G001682) and *CCR* (Pag.A10G000187 and Pag.A10G001433) genes increased under salt stress in 84K poplar. Most of *CAD* (Pag.A01G005119, Pag.A01G005123, Pag.A01G005202, Pag.A01G005205, Pag.A01G005239, Pag.A11G001707, Pag.A11G001727, Pag.A11G001731, Pag.A11G001742, Pag.A11G001754, Pag.A11G001755, Pag.B01G004751, Pag.B01G004773, Potri.011G163000, Potri.011G162884, Potri.011G162916, Potri.011G162828, Potri.011G162900, Potri.011G158100, Potri.011G155900, Potri.011G158901, Potri.001G459100, Potri.011G160700, Potri.011G161400, Potri.011G162732, Potri.001G463300, Potri.001G464700) genes increased under salt stress in both 84K and XYY. *4CL* (Potri.003G188500) and *CCR* (Potri.018G100500, Potri.006G178700, Potri.001G1440700) genes increased in XYY (**Figure 5**).

**Figure 5:**
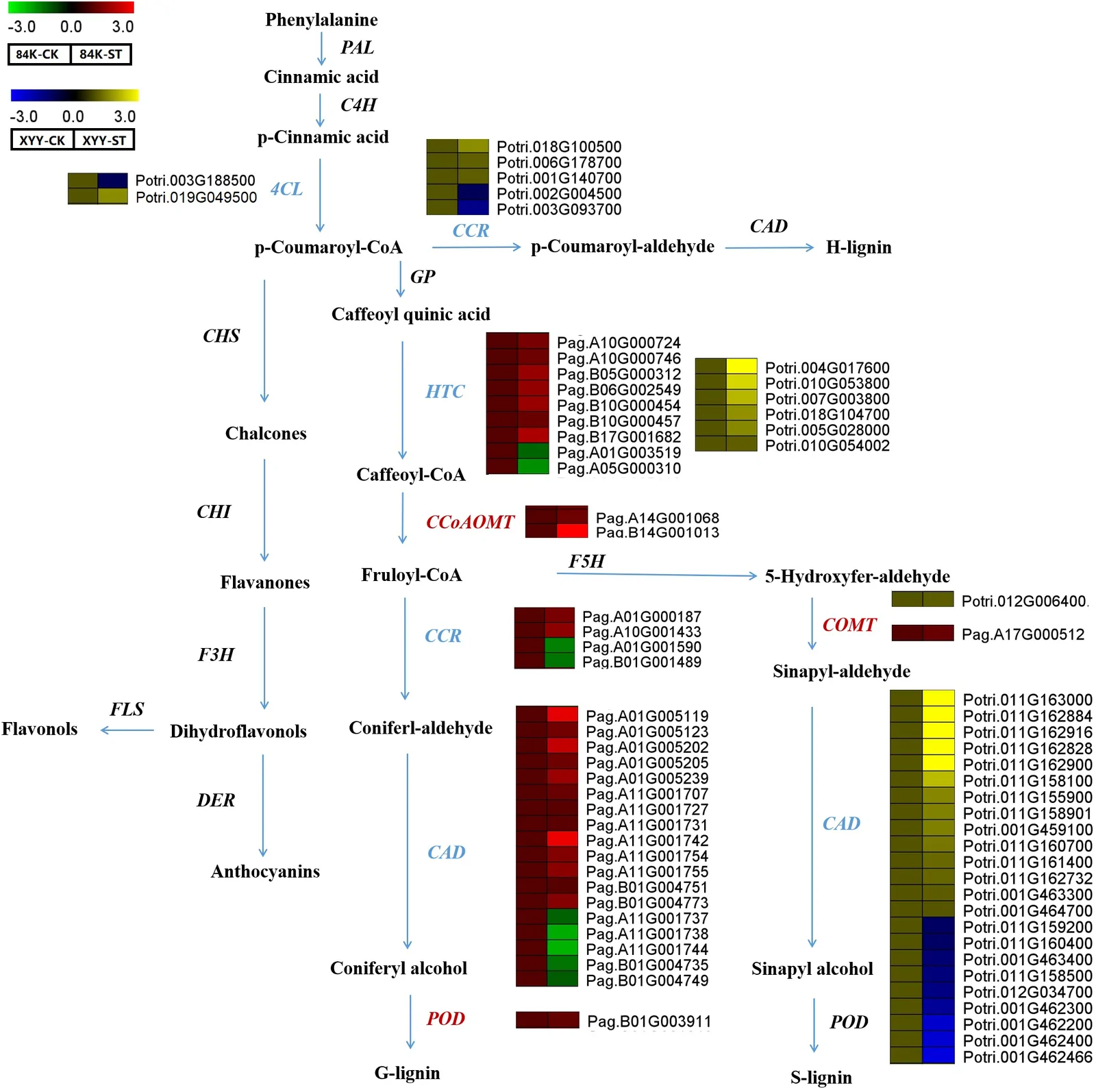
The phenylpropanoid biosynthesis pathway under salt stress in *Populus roots*. The boxes in the pathway represent DEGs. Red and green represent upregulated and downregulated genes in 84K. Yellow and blue represent upregulated and downregulated genes in XYY.

The key genes associated with casparian strip formation were screened in DEGs, including *MYB36*, casparian strip membrane protein 1 (*CASP1*) and peroxidase 64 (*PER64*). The results showed that *MYB36* expression was downregulated in 84K, while its expression was upregulated in the XYY. *CASP1* and *PER64* genes exhibited increased expression in both 84K and XYY poplar genotypes (**Table 1**).

**Table 1:** Genes related to the formation of casparian strip in poplar.

Gene name	Description	Gene ID	log_2_FC
*PaMYB36*	transcription factor MYB36 [Populus alba]	Pag.A18G000621.v3.1	-1.09
*PaMYB36*	transcription factor MYB36 [Populus alba]	Pag.B06G001132.v3.1	-1.11
*PtMYB36*	transcription factor MYB36 [Populus trichocarpa]	Potri.018G095900.v4.1	1.76
*PtMYB36*	transcription factor MYB36 [Populus trichocarpa]	Potri.006G171184.v4.1	1.22
*PtMYB36*	transcription factor MYB36 [Populus trichocarpa]	Potri.006G170800.v4.1	1.02
*PaCASP1B1*	CASP-like protein 1B1 [Populus alba]	Pag.A11G001691.v3.1	1.77
*PaCASP1B2*	CASP-like protein 1B2 [Populus alba]	Pag.A01G004850.v3.1	1.29
*PtCASP1*	casparian strip membrane protein 1 [Populus trichocarpa]	Potri.006G208800.v4.1	1.27
*PtCASP1B1*	CASP-like protein 1B1 [Populus trichocarpa]	Potri.011G154900.v4.1	1.07
*PaPER64*	peroxidase 64-like [Populus alba]	Pag.A05G001136.v3.1	3.62
*PtPER64*	Peroxidase 64 [Populus trichocarpa]	Potri.005G108900.v4.1	1.39

### 3.6 The starch and sucrose metabolism pathway in Populus roots under salt conditions

Under salt stress, the starch and sucrose metabolism pathway is also the main enriched pathway in 84K and XYY. The expression levels of most DEGs involved in the starch and sucrose metabolism pathway have increased. The expression levels of hexokinase (*HK*, Pag.A01G002940 and Pag.B01G002698), invertase (*TNV*, Pag.B03G000328), *SUS* (Pag.A17G000673, Pag.A19G000166, Pag.A19G000696, Pag.A19G000700, Pag.B12G000641, Pag.B17G000584, Pag.B19G000795, Pag.B19G001005, Pag.B19G001325, Pag.B19G001331 and Pag.B19G001336), trehalose-6-phosphate phos phatase (*TPP*, Pag.A02G001783, Pag.A08G001616 and Pag.B08G001524) and alpha-amylase (*AMY*, Pag.A16G001166) genes have all increased. *Glutamine synthetase* (*GS*, Pag.B10G002243) and glycogen phosphorylase (*GP*, Pag.B03G001876) genes decreased in 84K poplar roots under salt stress. The terpene synthase (*TPS*, Potri.012G078500), 1,4-alpha-glucan branching enzyme glgB (*glgB*, Potri.001G359200), glucose-1-phosphate adenylyltransferase (*glgC*, Potri.004G1157100, Potri.007G146100, Potri.014G110000) and most invertase (*INV*, Potri.015G127100, Potri.016G077400, Potri.016G077500) genes increased in XYY roots under salt stress (**Figure 6**).

**Figure 6:**
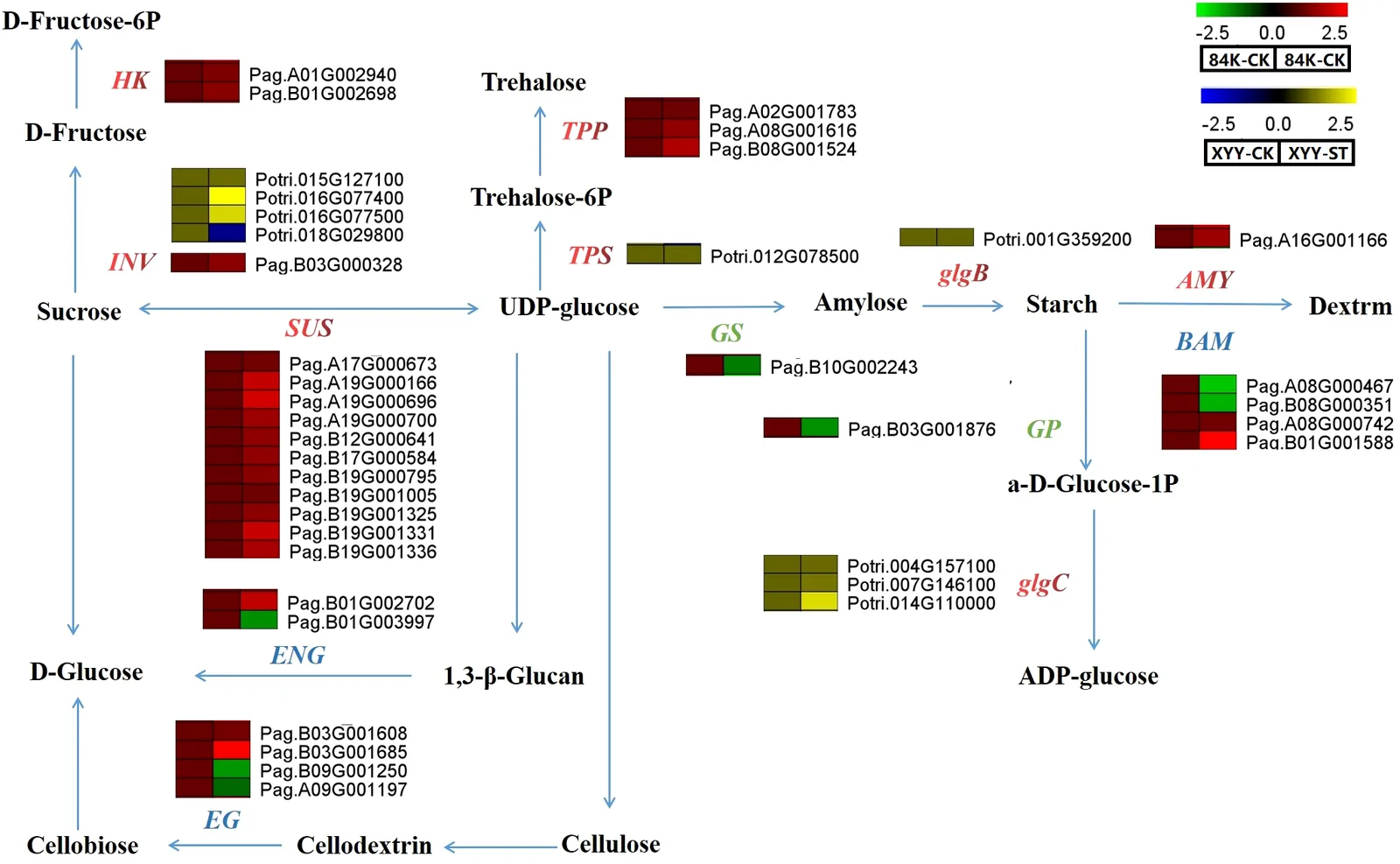
The starch and sucrose metabolism under salt stress *in Populus roots.* The boxes in the pathway represent DEGs. Red and green represent upregulated and downregulated genes in 84K. Yellow and blue represent upregulated and downregulated genes in XYY.

### 3.7 MAPK signaling pathway in Populus roots under salt conditions

According to the correlation analysis of the transcriptome in 84K and XYY roots under salt conditions, MAPK signaling pathway-plant was the main regulatory pathways under salt stress. The expression levels of protein phosphatase type 2C (*PP2C*, Pag.A01G002829, Pag.A09G000383, Pag.B01G002590, Pag.B08G001866, Pag.B09G000361, Pag.B12G000349, Potri.006G232700 and Potri.012G002700) were increased in 84K and XYY roots. Mitogen-activated protein kinase kinase kinase 17/18 (*MAPKKK17/18*, Pag.A01G003207, Pag.A04G000096, Pag.A09G000724, Pag.A11G000233, Pag.B04G000082 Potri.007G096400, Potri.002G228200, Potri.003G184200, Potri.005G139300, Potri.007G044800, Potri.009G073200, Potri.014G155000, Pag.A01G003207, Pag.A04G000096, Pag.A09G000724, Pag.A11G000233, Pag.B04G000082, Potri.001G142500, Potri.008G073400 and Potri.010G183900) and abscisic acid receptor PYR/PYL family (*PYR/PYL*, Pag.A06G001980, Pag.A08G001798, Pag.B03G001121 and Pag.B08G001715) genes were decreased in both 84K and XYY plants. After salt stress, *MPK4/6* (Pag.A14G000867) gene in 84K and *MEKK1* (Potri.004G140000) gene in XYY were upregulated (**Figure 7**).

**Figure 7:**
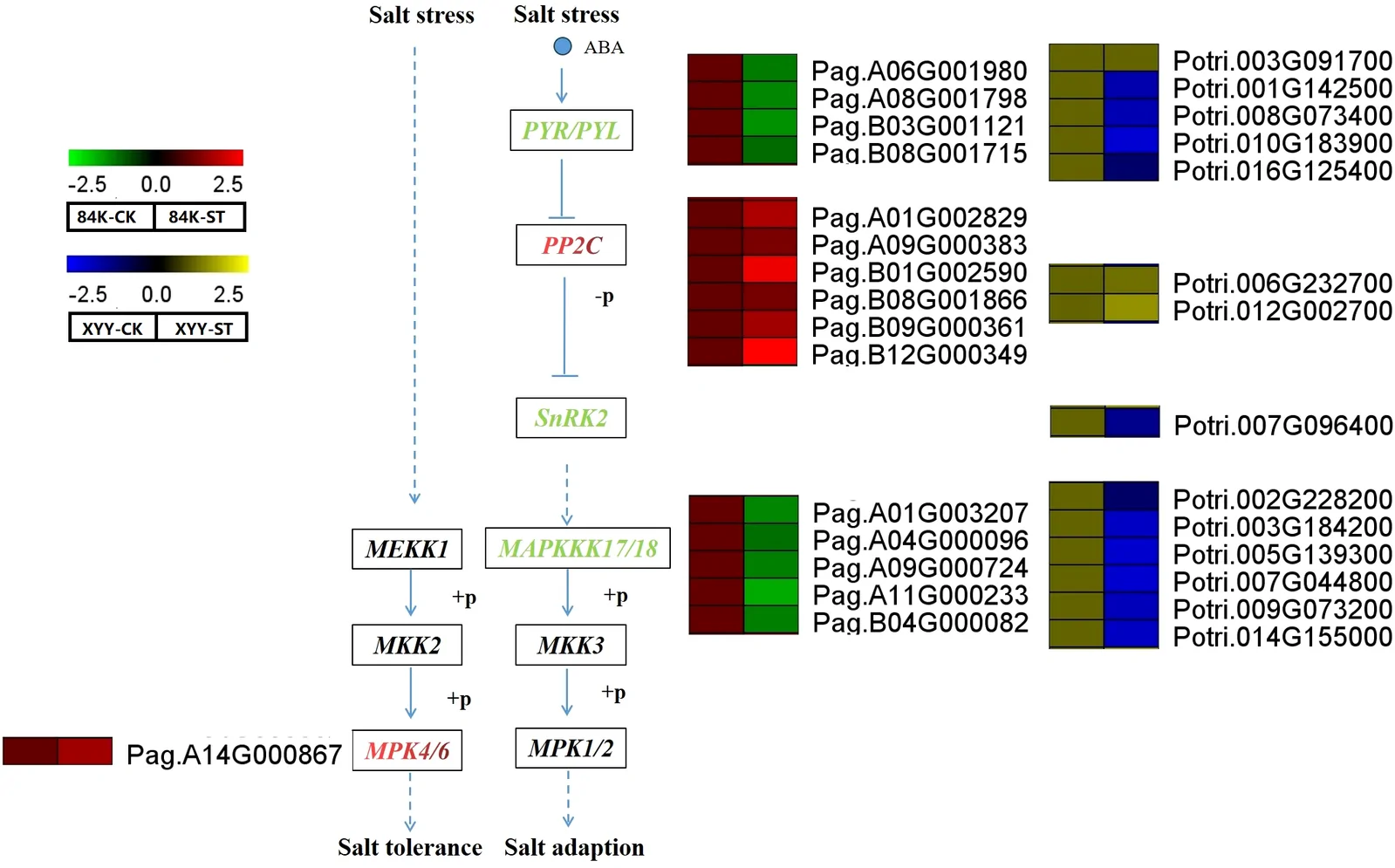
MAPK signaling pathway under salt stress in *Populus* roots. The boxes in the pathway represent DEGs. Red and green represent upregulated and downregulated genes in 84K. Yellow and blue represent upregulated and downregulated genes in XYY.

## 4 Discussion

### 4.1 Analysis of the response differences in lignin deposition in the roots of different poplar species under salt stress

The root is an important plant organ that is directly exposed to the soil, therefore, the effects of salinity stress are more pronounced in roots. The salt tolerance of the plant depends decisively on the tolerance of roots (Jin et al., 2024). Many researches had found that the lignin content in roots was related to salt resistance. In plant roots, the casparian strips also functions as a barrier to water and ion movement through the apoplast (Krishnamurthy et al., 2009).

Lignin is a primary constituent of the plant secondary cell wall and plays a crucial role in plant stress resistance; abiotic stress can rapidly induce its synthesis and deposition within the secondary cell wall (Cesarino, 2019; Vanholme et al., 2019). Lignin content accumulates in six poplar species under salt stress, especially in 84K poplar, lignin content increased significantly following salt treatment, reaching approximately 1.78 times the pre-treatment levels. Salinity altered the makeup of the cell wall by elevating the lignin-to-carbohydrate proportion in both salt-sensitive (*Populus canescens*) and salt-resilient (*Populus euphratica*) species (Janz et al., 2012). Similar observations have been reported in tomatoes, where the expansion of xylem and phloem cell areas contributes to the salinity tolerance of the plant (Chattha et al., 2024).

Lignin is synthesized from phenylalanine through the phenylpropanoid pathway, including the reactions catalyzed by PAL, 4CL, CAD, POD, CCR, COMT and CCoAOMT (Vanholme et al., 2019). Under salt stress, lignin content in maize seedling roots increases significantly. Compared to seedlings grown under normal conditions, the expression levels of lignin biosynthesis-related genes, *COMT1* and *CCoAOMT1*, are markedly up-regulated (Oliveira et al., 2020). In *Sesuvium portulacastrum*, salt-induced lignin accumulation in the roots is driven by the up-regulation of key biosynthetic genes—including *4CL*, *CAD*, *CCR*, *COMT* and *CCoAOMT* (Li et al., 2023). KEGG enrichment analysis of DEGs in 84K and XYY revealed a significant involvement of the phenylpropanoid biosynthetic pathway under salinity. Specifically, key genes such as *COMT* and *CCoAOMT* exhibited substantial up-regulation in response to the stress. Furthermore, lignin deposition in the roots has been identified as a critical component of the salt-tolerance strategy in chinese cabbage (Cao et al., 2020). CAD is a vital enzyme at the late stage of lignin biosynthesis (Vanholme et al., 2019). *ZmCAD1* exhibited a sustained up-regulation in response to salt stress (Zhao et al., 2026). Most *CAD* genes in 84K and XYY poplar exhibit positive regulation in response to salinity. The lignin content of six types of poplar trees increased under salt stress, which might be related to the enhanced expression levels of key genes in the phenylpropanoid pathway.

### 4.2 Analysis of the response differences of the casparian strips in poplar root systems under salt stress

The deposition of lignin in the root endodermis results in the formation of the casparian strips. The casparian strips, located within the endodermal and exodermal cell walls of vascular plant roots, serve as essential barriers that regulate nutrient selectivity, exclude salts and pathogens, and manage the transport of water and assimilates (Julkowska and Testerink, 2015; Song et al., 2019).

The relationship between casparian strips development and root growth was initially investigated in the genus *Calocedrus* (Wilcox, 1962). Results indicated that the distance from the root tip to the onset of the casparian strips decreases under osmotic stress. Similarly, Reinhardt and Rost (1995) proposed that salinity accelerates the maturation of the casparian strips, observing a reduction in the gap between the root apex and the most distal point of the strip during salt exposure. Our results demonstrate that salt stress accelerates casparian strip formation in poplar roots, this positional shift likely underpins the robust salt resistance of poplar, consistent with findings in rice, cotton, and maize (Krishnamurthy et al., 2009).

Under control conditions, the lignin content in 84K, QY and 717 was relatively low with no significant differences among them; furthermore, their casparian strips were located further from the root apex, and the degree of cell lignification was minimal. However, following salt stress, these three genotypes exhibited a significant surge in root lignin content and a substantial shift of the casparian strip toward the root tip. These results suggest that these varieties enhance cell lignification via the lignin deposition pathway to mitigate salt-induced damage. In contrast, NL895, XYY and FB3 maintained higher basal lignin levels and casparian strips closer to the root apex under normal conditions and showed negligible changes in lignin content and casparian strip positioning post-stress, these genotypes may be in a “preadapted” state, employing a constitutive defense strategy rather than an inducible strategy (Acosta-Motos et al., 2017).

In *Arabidopsis thaliana*, MYB36 can directly regulate the expression of most of the downstream genes involved in the formation of the chloroplast envelope, including *CASP1* and *PER6* (Kamiya et al., 2015). CASP1 is the organizer of a membrane platform and can guide the correct assembly of lignin biosynthetic enzymes (Roppolo et al., 2011). PER64 can catalyze the reaction between lignin monomers and H_2_O_2_ produced by respiratory burst oxidase homolog F, thereby promoting the polymerization of lignin (Lee et al., 2013). The expression levels of *CASP1* and *PER64* genes in poplars were both increased, which might also be the reason for the increase in lignin under salt stress. Interestingly, the expression level of *MYB36* decreased in 84K and increased in XYY, which is consistent with the phenotype that casparian strips form farther from the root tip in 84K but closer in XYY. Differential expression of the *MYB36* gene may serve as the molecular mechanism responsible for this developmental difference (Touqeer et al., 2026).

### 4.3 The starch and sucrose metabolism pathway in populus roots under salt conditions

Plants utilize carbohydrate accumulation, specifically sucrose, to achieve osmotic homeostasis (Nie et al., 2022) and to direct the multi-layered regulatory networks that control root growth during salt exposure (Zhang and Zhang, 2022). Regarding the KEGG pathway enrichment analysis, starch and sucrose metabolism DEGs in response to poplar roots under salt stress. An upregulated expression of sugar reducing and starch synthesis and degradation genes was also observed in the roots of *T. chinensis* (Li et al., 2023).

The enzymatic processing of sucrose, mediated by TPP, SUS, HK, glgB, glgC and AMY, acts as a metabolic conduit, providing the essential precursors for the biosynthesis of proteins and structural polymers such as starch, cellulose, and callose. Beyond these biosynthetic functions, sucrose metabolism operates as a signaling network that coordinates plant developmental pathways (Duan et al., 2021). The expression of the *TPP* gene in 84K poplar was up-regulated under salt stress. A similar *TPP* gene expression pattern was found in grape (Chen et al., 2025), and wheat where significant up-regulation of the *TaTPP1/2/4/9* transcripts was observed at the stage of salt treatment (Islam et al., 2021).

The regulation of cellulose synthesis and salt tolerance is closely linked to *SUS* activity. Amor et al. (1995) found that plasma membrane-localized *SUS* facilitates carbon channeling into cellulose biosynthetic complexes in cotton fibers. This mechanism is consistent with findings in poplar, where *PsnSUS2* overexpression enhances cellulose accumulation and cell wall density (Wei et al., 2015), and *PtrSUS3/5* transcript levels align with cellulose production (Zhang et al., 2023). Beyond structural reinforcement, *SUS* isoforms like *ZmSUS1* also mediate physiological resilience to salinity by reconfiguring sucrose metabolism and osmotic balance (Xiao et al., 2024). The increased expression of the *SUS* gene in 84K poplar may lead to elevated cellulose content, thereby contributing to enhanced salt tolerance. Similar to the salt-induced up-regulation of *HK* and *INV* observed in *Dendrobium officinale* (Hao et al., 2024), 84K and XYY poplar genes encoding these enzymes also displayed enhanced expression during salt exposure.

Starch breakdown products are critical for sustaining plant ontogeny and enhancing resilience to environmental stressors (Thalmann and Santelia, 2017; Yang et al., 2023). As an endo-hydrolase, AMY catalyzes the cleavage of α-1, 4-glycosidic linkages within polysaccharides, yielding a suite of oligosaccharides and sugars, including dextrin, maltotriose, maltose, and glucose (van der Maarel et al., 2002; Farooq et al., 2021; Zhang and Yang, 2023). Salinity-triggered up-regulation was observed for *StAMY6, 14, 16, 17, 19 and 20*, highlighting their likely involvement in mediating salt stress tolerance. This suggests a coordinated role for these isoforms in the physiological response to high salinity (Duan and Jin, 2024). The *AMY* gene is upregulated in hyacinth bean under salinity tolerance (Srinivasa and Rangaiah, 2015). Like potatoes and Hyacinth beans, the *AMY* gene in poplar trees also positively responds to salt stress. The alterations in key genes within the starch and sucrose metabolic pathways of poplar roots under salt stress may also represent a critical mechanism underlying lignin accumulation, thereby enabling poplar to cope with salt stress.

### 4.4 MAPK signaling were associated with salt tolerance in poplar roots

MAPKs are critical signaling proteins that relay messages from various environmental stressors and developmental signals, serving as a bridge between stimulus perception and cellular response (Smékalová et al., 2014). Heightened salt resilience in rice is achieved through the overexpression of *OsMKK6*, which manifests as superior root and shoot development, including increased biomass and length, when subjected to salinity (Kumar and Sinha, 2013). Along with roles of MPK4 and MPK6 in various stress responses, they are involved in multiple developmental processes. Thus, MPK6 is involved in embryogenesis (Bergmann et al., 2004), cell division plane orientation and root development (Smékalová et al., 2014). MPK4 controls cell division, root growth and formation of root hairs (Beck et al., 2011). Roots of *mpk4* and *mpk6* mutant effect on root hair morphogenesis and hydrogen peroxide accumulation in *Arabidopsis* (Takáč et al., 2016). The expression level of *MPK4/6* was elevated in 84K poplar under salt stress, but there was no change in XYY poplar. This might be one of the reasons for the higher lignin accumulation in 84K poplar.

SnRK2 plays a crucial regulatory role in plant resistance to abiotic stress. Most of the rice and *Arabidopsis* SnRK2 kinases are activated by salt treatment (Boudsocq et al., 2004; Kobayashi et al., 2004). MpSnRK2.10 can alleviate the salt stress inhibition of apple by regulating Na^+^ balance and ABA pathway (Lou et al., 2018). SnRK2 interact with and phosphorylate AtNST1 enhances plant secondary cell-wall formation and lignin deposition in the stem fiber region (Liu et al., 2021). The expression level of SnRK2 in XYY was decreased, while that of 84K remained unchanged under salt stress, this might have led to a relatively lower accumulation of lignin in XYY.

The PP2C family consists of critical signaling components that modulate numerous biological pathways, playing a decisive role in the regulation of plant responses to abiotic stress (Fuchs et al., 2013). The expression levels of *PP2C* genes in alfalfa roots were up-regulated under salt stress (Wang et al., 2023). Both *AhPP2C45* and *AhPP2C134* emerge as promising *PP2C* candidates that may mediate salinity resilience in peanuts (Wu et al., 2023). The *BpPP2C1* gene exhibited high levels of transcriptional activation in the roots of *Betula platyphylla* under salt-stressed conditions (Xing et al., 2020). The expression of *PP2C* genes was also up-regulated in poplar roots, indicating their involvement in the regulation of salt tolerance.

PYR/PYL controlling ABA signaling by inhibiting *PP2C* (Cutler et al., 2010). Under salt stress, the expression levels of most *PYR/PYL* genes in poplar decreased, while *PP2C* genes were up-regulated, this regulatory pattern likely contributes to the salt tolerance of the poplar. MAPK pathways are essential for facilitating salt-stress signaling and tolerance mechanisms in poplar.

## 5 Conclusion

Lignin enrichment and changes in the casparian strip in different poplars roots under salt stress. *P. alba × P. glandulosa* ‘84K’ showed the most pronounced changes, followed by *P. cathayana, P. tremula × P. alba*, *P*. *deltoids* × *P*. *euramericana* ‘Nanlin895’, *Populus* × *euramaricana* ‘Bofeng 3 hao’ and *P. simonii.* Transcriptome sequencing revealed the molecular mechanisms in the 84K poplar and and *P. simonii* with the greatest and smallest lignin increase and both of them through phenylalanine metabolism, starch and sucrose metabolism, and MAPK signaling pathway. This study provides critical theoretical support for elucidating the molecular response mechanisms of root lignin and the casparian strip under salt stress, laying a foundation for the molecular breeding of salt-tolerant varieties.

## Data Availability

The data presented in the study are deposited in the NCBI database, accession numbers are PRJNA1494161 and PRJNA1494114.
