# Supplementary material for: The lignin deposition responses of different poplar root systems to salt stress
Source: Front Plant Sci. 2026 Sep 22;17:1888861. doi: 10.3389/fpls.2026.1888861 (PMC13638378; doi:10.3389/fpls.2026.1888861)
Supplement: Supplementary file 1 [file DataSheet1.docx]

**Figure S1.** (A) Principal component analysis of expressed genes. (B) Distribution of gene expression in CK and ST. (C) Volcano map of DEGs in 84K *Populus* roots under salt stress. (D) The heatmap of DEGs in 84K *Populus* roots under salt stress.

**A**

**B**

**C**

**D**

**Figure S2.** (A) Principal component analysis of expressed genes. (B) Distribution of gene expression in CK and salt stress. (C) Volcano map of DEGs in *Populus simonii* roots under salt stress. (D) The heatmap of DEGs in *Populus simonii* roots under salt stress.

**Figure S3.** qRT-PCR validation of the 84K poplars roots differentially expressed genes in salt treatments.

**Figure S4** Validation of the differentially expressed genes in *Populus simonii* roots under salt stress using qRT-PCR.

**Table S1.** The primers used for qRT-PCR.

| Gene | | Forward | Reverse |
| --- | --- | --- | --- |
| *Pag.A06G001980* | 5' CGCCGGGAAATACTAAAGAGG 3' | | 5' GATAGCGACAGCGATGGAAAA 3' |
| *Pag.A01G002829* | 5' GCGATTCACCCTTCATTCTGT 3' | | 5' ACTTCCTTGTCCATCCTCCGA 3' |
| *Pag.B01G002590* | 5' GCGATTCACCCTTCATTCTGT 3' | | 5' ACTTCCTTGTCCATCCTCCGA 3' |
| *Pag.B03G000328* | 5' ACAAAATCAGTGGCGGTGGT 3' | | 5' TGTGGCGTATAAGAGTCGGG 3' |
| *Pag.B16G001443* | 5' TTACTGGGGAGGTGGGTTGG 3' | | 5' CTCGGAGGGGTTCCTTTATA 3' |
| *Pag.B19G001336* | 5' CTCAACTCAAACGGCTCACC 3' | | 5' CCACCTCGCCAATAACAATG 3' |
| *Pag.B01G001588* | 5' AGAATCAGCAATAGGAGTCAA 3' | | 5' CCCACAAATCAGAAGCATCAG 3' |
| *Pag.A09G000097* | 5' CTTCTATCCTAGGGGGGGGCT 3' | | 5' GGCGTGAATGTGGCATAATCC 3' |
| *Pag.A17G000069* | 5' CTCAACTCAAACGGCTCACCT 3' | | 5' CCAACCCACCTCGCCAATAAC 3' |
| *Pag.A05G001473* | 5' GTCAGAAGGTGGTCGGAACG 3' | | 5' TGGGGACTGTAGGAGAGGCA 3' |
| *PagActin* | 5' ACCCTCCAATCCAGACACTG 3' | | 5' TTGCTGACCGTATGAGCAAG 3' |
|  |  | |  |
| *Potri.004G140000* | 5' TACTTGGCTGTCCTTGTGTTA 3' | | 5' TCATCTTCTCTTTGCTTTTGG 3' |
| *Potri.001G142500* | 5' GGGTTTAGTATTATTGGAGGA 3' | | 5' ACAGACGCAAGTTTTTGGAGA 3' |
| *Potri.010G183900* | 5' CGGTCTGTTACCACGCTTCAT 3' | | 5' GGGCATTCCTCGCCATTTTCT 3' |
| *Potri.006G232700* | 5' TGGGAATCTGCATATCCTCTGCAT 3' | | 5' ATGCAGAGGATATGCAGATTCCCAT 3' |
| *Potri.007G044800* | 5' GAGTGGGTAAGAGGAGGCTGCC 3' | | 5' GGCAGCCTCCTCTTACCCACTCCAT 3' |
| *Potri.005G100400* | 5' AGAGGTGCGAGTGCTAAAGGG 3' | | 5' CGAATGAAGAAGACTGGGAAA 3' |
| *Potri.002G088600* | 5' GATGAAGGAACGGCTGATAAG 3' | | 5' GCAAACTAGAGTGACAGGGAG 3' |
| *Potri.017G131100* | 5' ATAAGGAGCAAAGGAAGGACT 3' | | 5' GTATCGCAAATGACAAGGATA 3' |
| *Potri.009G008600* | 5' GTTCGATCTAAAGCTTCACAC 3' | | 5' GCCAAATACAGAAAACTCCTC 3' |
| *Potri.012G113500* | 5' ACATGAGAGAGGTGGGAAAGT 3' | | 5' AAAGCACACGGGATAAGGACA 3' |
| *PtrActin* | 5' AGGCAGGTTTCGCAGGAGATGA 3' | | 5' TCCATCACCAGAATCCAGCACA 3' |

**Table S2.** Summary of the sequencing data generated for RNA-seq and mapping of the 84K *Populus* genome.

| Sample | Raw Reads | Clean Reads | GC Content | %≥Q30 |
| --- | --- | --- | --- | --- |
| CK-1 | 20,875,557 | 6,247,895,744 | 44.32% | 95.57% |
| CK-2 | 24,007,595 | 7,183,966,080 | 44.30% | 96.00% |
| CK-3 | 255,963,151 | 6,874,403,078 | 44.31% | 95.46% |
| ST-1 | 22,928,689 | 6,865,068,002 | 44.28% | 95.55% |
| ST-2 | 22,725,122 | 6,802,152,722 | 44.37% | 95.75% |
| ST-3 | 20,283,063 | 6,071,961,172 | 44.31% | 95.41% |

**Table S3.** Summary of the sequencing data generated for RNA-seq and mapping of the *Populus simonii* genome.

| Sample | Raw Reads | Clean Reads | GC Content | %≥Q30 |
| --- | --- | --- | --- | --- |
| CK-1 | 21,780,729 | 6,512,867,934 | 44.13% | 95.47% |
| CK-2 | 23,182,324 | 6,938,811,382 | 44.10% | 94.87% |
| CK-3 | 26,262,424 | 7,856,588,000 | 44.17% | 94.77% |
| ST-1 | 21,846,693 | 6,540,449,844 | 44.26% | 94.58% |
| ST-2 | 24,014,591 | 7,187,279,802 | 44.25% | 94.71% |
| ST-3 | 22,072,272 | 6,609,231,354 | 44.15% | 94.45% |
